# The Role of Father Involvement and Marital Satisfaction in the Development of Family Interactive Abilities: A Multilevel Approach

**DOI:** 10.3389/fpsyg.2016.01725

**Published:** 2016-11-07

**Authors:** Alessandra Simonelli, Micol Parolin, Chiara Sacchi, Francesca De Palo, Alessio Vieno

**Affiliations:** Department of Developmental and Social Psychology, University of PadovaPadova, Italy

**Keywords:** early family interactions, father involvement, dyadic satisfaction, multilevel analysis, developmental trend

## Abstract

The study aims to investigate the development of family interactions from pregnancy to preschool age in a longitudinal perspective, using multilevel analysis. Also, it explored the impact of couple relationship and father involvement in childcare on the developmental trend of the quality of mother–father–child interactions. One hundred and three primiparous families were assessed at 7th month of pregnancy, 4th, 9th, and 18th months of child’s life and during preschool age (36–48th), using the observational procedure named, Lausanne Trilogue Play. Parents’ perception of marital satisfaction was assessed with the Dyadic Adjustment Scale at each point of measure; moreover, in the postnatal assessment, parents completed the Father Involvement Questionnaire. Results showed that family interactions increase over time. Secondly, a decrease of marital adjustment is associated with an improvement of the quality of family interactions. Moreover, father involvement predicts the quality of family interactions from the earliest stages of child’s life. In a longitudinal perspective, family interactions and marital quality show opposite developmental trends and father’s involvement represents a particularly important feature of the family.

## Theoretical Background

### Early Family Interactions

This study used a growth modeling approach to investigate family interactions from prenatal to preschool age, including the examination of predictors of their trajectories, namely, marital satisfaction and father involvement.

In the last 30 years, studies in the framework of Infant Research have recognized the importance of early mother–child interactions on child’s development; however, most of them have applied a dyadic perspective. Little research has focused on mother–father–child triad and has used longitudinal designs, aimed at examining developmental family trajectories.

A particularly important approach to the study of early family interactions is the one proposed by Corboz-Warnery et al. (1993), providing both a theoretical framework and a methodological model to the study of family system. At the theoretical level, this approach conceptualizes the importance of the *primary triangle*, constituted by mother–father–child interactions, and it states that this triad is not a mere extension of the dyadic system (mother–baby or father–baby), but, rather, it develops according to specific and unique pathways (Fivaz-Depeursinge and Corboz-Warnery, 1999). In this view, early family interactions constitute an early developmental matrix for child’s affective and relational development, in terms of intersubjective competences (Fivaz-Depeursinge and Corboz-Warnery, 1999; Fivaz-Depeursinge, 2002; Fivaz-Depeursinge et al., 2005, 2010; McHale et al., 2008). Besides primary and secondary intersubjective abilities, research underlines child’s ability to interact with more than one partner simultaneously, at an earlier stage than the ninth month of life (Nadel and Tremblay-Leveau, 1999; Striano et al., 2007; Tremblay and Rovira, 2007). In fact, empirical studies (Fivaz-Depeursinge et al., 2000, 2005; Frascarolo et al., 2004) have shown that babies manifest indicators of coordination of attention and affection toward both parents, while interacting with them together, already at 3–4 months. Hence, the Lausanne model claims that child’s capacity to interact with two partners develops concurrently and not subsequently to dyadic competences (Fivaz-Depeursinge et al., 2010).

According to these empirical and theoretical backgrounds, authors have ideated and implemented an observational procedure, the Lausanne Trilogue Play (LTP, Fivaz-Depeursinge and Corboz-Warnery, 1999), which was specifically designed with the purpose of observing and assessing the quality of mother–father–infant/child interactive competences and infant early intersubjectivity abilities. It is a semi-structure observational procedure in which parents are asked to play with their child; it enables to observe the actual interactions that occur in the family system after childbirth. Moreover, thanks to its prenatal version (Prenatal LTP; Carneiro et al., 2006), it permits the examination of the co-parental interactive behaviors during pregnancy, in accordance with the definition of co-parenting as the quality of the coordination presented by the interactive exchanges between two parents while they are taking care of their child (Minuchin, 1974; McHale, 1995). Continuity has been attested between expectant parents’ exchanges and postnatal co-parental interactive features, and prenatal interactions are recognized as one of the most important predictors of later family interactions’ quality (Carneiro et al., 2006; Simonelli et al., 2012; Favez et al., 2013). Thus, co-parental abilities during pregnancy can be conceived as an interactive matrix for the construction of early family relationships.

Regarding the development and change of the quality of triadic interactions over the postnatal period, little research has specifically investigated this domain adopting the LTP procedure and the available results are partially inconsistent. A first study supported the stability of family interactions quality over the first year and until the 18th month of child’s life (Weber, 2002); in addition, no significant differences were detected from pregnancy to toddlerhood (Carneiro et al., 2006; Favez et al., 2006a). However, other studies (Favez et al., 2006b, 2012), whilst confirming the stability of family interactions’ quality, reported the presence of three different patterns of development for family interactive coordination. In fact, although two groups of families showed a stable pattern (with high and low stability, respectively), results have identified a third group of families characterized by a decline in triangular interactive quality, specifically from the 5th month of pregnancy to the 18th month of child’s life; both marital and child’s characteristic were implicated in differentiating the longitudinal trends. In addition to the stability and declining models, other results, obtained from an Italian sample, indicated a different pattern of development of early triangular interactions, with an improvement of their quality from pregnancy to the postnatal period, mainly occurring at fourth and at ninth month of child’s life (Bighin et al., 2011).

Overall, the available literature on the development of family interactions shows some limitations. To date, the stability of triangular interactive quality from pregnancy onward is still debatable: some authors support the absence of change in family interactions during the early postnatal years, while other studies indicate different possible patterns of development. However, the number of studies adopting a longitudinal approach is restricted, especially considering the preschool age: if considerable attention has been paid to the first year after birth, little is known about toddlerhood and, even at a smaller extent, preschool age.

### Parental Dimensions Related to Family Interactions

Among the various factors able to shape parental functioning (Belsky, 1984), paternal involvement and marital satisfaction have been largely demonstrated to be strictly associated to family relationships. For this reason, this study focused on the examination of father involvement in childcare and of dyadic satisfaction, interpreting them as dimensions related to the quality and/or the development of family interactions.

The construct of Paternal Involvement, offered by Lamb et al. (1985) represents a valuable perspective for the analysis of father’s participation in childcare. According to author’s description, paternal involvement is composed of three factors: accessibility, that is physical presence, even if not directly involved in shared situations/activities with the child; responsibility, concerning taking decisions about childcare, health and education; engagement, which includes direct interactions between father and child in play situations and daily-care activities. Although father involvement is mainly experienced in triadic interactive context rather than in dyadic situations (Belsky and Volling, 1987), a small number of studies have specifically assessed father’s role in triadic interactions. A first study, applying the LTP, reported a significant correlation between prenatal father’s representation of his future role and of child’s characteristics and the quality of child interactive competences, considering both dyadic (father–child) and triadic (father–mother–child) contexts, over the first year of life (Von Klitzing et al., 1999). Also Frascarolo (2004) highlighted that father involvement in childcare affects the whole family system. In contrast, other preliminary studies found no significant associations between fathers and mothers’ perception of father involvement and the quality of triadic interactions assessed with LTP at fourth month of child’s life (Simonelli et al., 2008). Finally, a study has suggested a pattern of change over time for father involvement; Coley and Chase-Lansdale (1999) observed that nearly 40% of unwed fathers either increased or decreased their level of involvement between child’s birth and his/her third year of life. This result may suggest that father involvement could differently exert its influence according to different developmental stages of the child or it can be differently influenced by child’s interactive competences.

The marital relationship has been commonly considered the most important family subsystems, establishing the basis for the emotional and relational functioning of the whole family and influencing family interactions during the transition to parenthood (Cowan and Cowan, 1992; Belsky and Kelly, 1995; McHale, 1995; Shapiro et al., 2000). Marital satisfaction is conceived as a construct subject to change, with specific trajectories over time, according to the different developmental transitions in the family life cycle. Despite some inconsistent results (Foran et al., 2013), it is widely recognized that marital satisfaction reaches the highest levels close to marriage and, after this, it shows a slow but constant decline until middle-age (Gottman and Notarius, 2002; Hirschberger et al., 2009; Mitnick et al., 2009). Within this general trend, partners are continuously asked to face family life cycle events that require change and put marital satisfaction at risk; transition to parenthood represents one of the most stressful challenges. Pregnancy and childbirth, in fact, punctuate the need for the couple to face changes (Belsky and Rovine, 1990; Cowan and Cowan, 2000) both at the inner and the behavioral levels (Cowan, 1991); in addition, there is also the need to reorganize the family, moving from a dyadic to a triadic system, in order to meet child’s need and acquire parenting competence. At the same time, parents have to buffer the potential adverse effects of these strains and difficulties on the marital relationship (Fivaz-Depeursinge and Corboz-Warnery, 1999). Since the 1980s, both cross-sectional and short-term longitudinal studies addressing the first baby’s birth have indicated that transition to parenthood is a critical period for marital satisfaction, which goes through a small but reliable decline (Belsky and Pensky, 1988; Belsky and Rovine, 1990; Cowan and Cowan, 1995; Twenge et al., 2003; Perren et al., 2005; Lawrence et al., 2008; Doss et al., 2009; Mitnick et al., 2009), despite a brief period of marital happiness immediately after childbirth, which is referred as “baby honeymoon” (Wallace and Gotlib, 1990). According to some authors, however, not all couples experience this transition as a challenging period, reporting a modest enhancement in their satisfaction (Belsky and Rovine, 1990; Cowan and Cowan, 1992; Levy-Shiff, 1994; Belsky and Kelly, 1995; Shapiro et al., 2000; Hudson et al., 2001; Kamp Dush et al., 2008; Anderson et al., 2010; Holmes et al., 2013). It can be assumed that there are different trajectories of change in marital satisfaction across transition to parenthood, with a trend that may be linear (Shapiro et al., 2000; Lawrence et al., 2008), curvilinear (Claxton and Perry-Jenkins, 2008) or different in the pre- and post-partum (Lawrence et al., 2007). A meta-analysis (Mitnick et al., 2009) and a 8-year longitudinal study (Doss et al., 2009) demonstrated that all newlywed couples face a decline over the years after marriage, whether they become parents or not, suggesting a general trend of decline, common to all couples and not merely specific to partners who face the transition to parenthood. Rather, becoming parents constitutes an additional stress that exacerbates the spontaneous and common decrease in marital happiness, making the decline steeper than before childbirth (Lawrence et al., 2007). With regard to the trajectories of martial satisfaction after childbirth in a long term perspective, empirical evidence suggested that the negative effect of transition persists during the preschool age, at 3 (O’Brien and Peyton, 2002), 4 (Doss et al., 2009), and 6 years of child’s age (Shapiro et al., 2000). The longitudinal study by Hirschberger et al. (2009) reported a decline for both partners until adolescence.

One of the most widely used instruments to assess marital satisfaction is Dyadic Adjustment Scale (DAS; Spanier, 1976); the existing studies on the transition to parenthood using DAS have focused prevalently on the trajectories of marital satisfaction from pregnancy to 3rd (Terry et al., 1991; Tomlinson, 1996), 4th (Shapiro and Gottman, 2005; Harwood et al., 2007), 6th (Wallace and Gotlib, 1990; Rholes et al., 2001; Van Egeren, 2004), 9th (Belsky et al., 1983), 12th (Elek et al., 2003; Condon et al., 2004), and 18th month post partum (Favez et al., 2006a,b, 2011, 2012; Trillingsgaard et al., 2012), overall indicating a decline in marital satisfaction. Only two studies examined changes in marital satisfaction over a longer period of time, at 24th (Kohn et al., 2012) and 30th month after birth (Trillingsgaard et al., 2014), confirming the decreasing trend; thus, there is a lack of empirical evidence investigating the trajectories of new parents’ marital satisfaction, assessed with DAS, up to the preschool age.

Regarding the association between marital satisfaction and family interactions in the context of the transition to parenthood, the available research on DAS and LTP do not display fully consistent results. On one side, studies (Favez et al., 2006a, 2013) reported the absence of a link between marital satisfaction and the quality of family interactions. On another side, an association has been detected between marital satisfaction in the prenatal period and the evolution of family interactions over the first 18 months of child’s life (Favez et al., 2006b; Darwiche et al., 2015), with high marital satisfaction predicting a decreasing pattern of family interactions. A negative correlation was also reported by a study conducted in 2011 (Favez et al., 2011), indicating that higher levels of marital satisfaction are associated with a lower quality of family interactions, in accordance with a new conceptualization of the decrease of marital satisfaction as a necessary and adaptive process for the transition from the dyadic system to the establishment of triadic family interactions. As recommended by Favez et al. (2013), further investigations are required in order to better clarify the nature of the connection between spouses’ satisfaction with their relationship and family interactions.

To date, only few studies have investigated the developmental trajectory of family interactions with the LTP and beyond the 18th month postpartum; in addition, results are non-univocal and investigations have not always included interactions predictors, such as father involvement and dyadic satisfaction. In the attempt to overtake the above-mentioned shortcomings and to clarify the nature of family interactions in the context of the transition to parenthood until the preschool age, the present study pursued two main objectives:

First, the research investigated the trend of the quality of triadic interactions from pregnancy to the preschool age (36–48 months). It was examined if early family interactions show a stable pattern during the transition to parenthood and beyond, or if they change over time. In particular, on the basis of previous literature, our hypotheses suggest an increasing trend of the quality of family interactions over time. Moreover, the study explored the developmental trends of dyadic satisfaction and father involvement, from pregnancy and the fourth month, respectively, over the first years of child’s life. According to literature, even though it is presumable to suggest that father involvement may increase over time, a declining trend of dyadic satisfaction is expected.

Second, a limited number of empirical studies have addressed the association of early family interactions with parents’ perception of father involvement in childcare and marital satisfaction; our objective was to investigate if the degree of father involvement and couple adjustment is related to the quality of family interactions and to their trend until the preschool age. It was postulated that higher levels of father involvement correspond to better triadic interactions, whereas regarding marital satisfaction, it is not clear the nature of this connection over time.

With respect to the methodology usually applied in longitudinal studies, the multilevel approach represents a promising method to analyze data, which, to our knowledge, has not been used before in the field of early triadic interactions and their development. In fact, empirical studies on family interactions with LTP usually adopt linear models as analytic strategy of data. However, neglecting the multilevel structure of the data may lead to incorrect inferences because of the underestimation of the standard errors of regression coefficients (Raudenbush and Bryk, 2002). Thus, the application of multilevel growth modeling can be used to overcome methodological limitations on the study of family interactions.

## Materials and Methods

### Procedure and Participants^1^

The longitudinal and multi-method approach used in this study consists of five stages: 7th month of pregnancy, 4th, 9th, 18th, 36/48th (referred as preschool age) month of child’s life. In each stage, families were assessed both with self-report measures and observational procedures, with the only exception of the 4th stage (18th month), when only self-report measures were administered.

For this study, data were collected between 2006 and 2012. Participants (*N* = 103 families) were non-referred primiparous families recruited during pregnancy at child’s birth preparation classes of the Obstetrics and Gynecological Clinic of Padua Hospital and received at the Department of Developmental Psychology and Socialization of the University of Padua for each stage. All families were asked to take part in five steps of the research, corresponding to five stages of child’s development.

Due to the panel attrition which inevitably characterizes longitudinal research (Ribisl et al., 1996; Tourangeau and Ye, 2009), 93.2% families were surveyed in the first stage (Pregnancy, *N* = 96), 84.46% at the second stage (4th month, *N* = 87), 73.78% at third stage (9th month, *N* = 76), 38.83% at fourth stage (18th month, *N* = 40), and 43.98% at fifth stage (preschool age, *N* = 41). A key advantage of the random-effects approach is its application when subjects are not measured at the same number of time points (Hedeker and Gibbons, 1997): this allows the researcher to successfully manage missing values. For this reason, the final samples (in which we have at least two time points for each respondent) was thus composed of 82 families.

Pregnancies and deliveries were medically uncomplicated; all infants were in good health and 59.8% were male. None of the parents presented a diagnosis of psychiatric disorders. Fathers’ age ranged from 26 to 54 years (*M* = 34.83, *SD* = 4.65), and mothers’ age ranged from 23 to 42 years (*M* = 32.67, *SD* = 3.94). The median level education was secondary school diploma, both for mothers and fathers (range = secondary school-university). The mean length of the couple’s relationship was 8 years (7.98), including both engagement and marriage years (*SD* = 4.41, range = 1.5–24 years).

Families which did not take part in the research (*N* = 7), due to a drop out in the first stage of the survey, presented no differences for socio-demographic data. In fact, father’s average age was 35 years (*SD* = 5.59, range 29–41), while mothers, were 31 years old (*SD* = 2.77, range 28–35) and the length of relationship was on average, 6.6 years (*SD* = 4.52, range: 2–14). With respect to education, also for these families, secondary school degrees represented the median level for both fathers and mothers.

### Methods of Data Collection

Each family was assessed with several self-report questionnaires and observational procedures:

– The *Questionnaire on Father Involvement* (Frascarolo, 1994, unpublished) is a 10-item questionnaire that assesses paternal participation in daily childcare activities, such as diapering, bathing, feeding, and taking baby to the pediatrician; items’ content varies according to child’s age. Each item represents a different activity with the child and can be scored from 0 to 2, according to the frequency of father’s management or participation. The Father Involvement total score results from the sum of each item score divided by the maximum number of points he could obtain, according to the number of items the subject answered to, converted to percentages. The ratio of the sum of scores and the sum of all the possible answers gives an overall score, defined as “variety index,” ranging from 0 to 100, with higher total scores corresponding to a higher father’s involvement in childcare activities. Father Involvement was assessed at 4th, 9th, 18th month, and at preschool stage. In the present study, the questionnaire was administered separately to fathers and mothers, in order to obtain both maternal and paternal perceptions of father’s involvement. For each step, the two evaluations were compared and, in case of no statistically different results, an index for each couple was calculated in terms of mean score of maternal and paternal measures, indicating the degree of father’s involvement according to the perception of the couple. In the present sample, the Father Involvement Questionnaire showed high reliability in each stages of the study: Cronbach’s alpha ranged from 0.66 to 0.84 for fathers’ reports and between 0.75 and 0.80 for mothers’ own.
– The DAS (Spanier, 1976; Italian version translated and validated by Gentili et al., 2002) is a 32-item self-report questionnaire assessing the degree of marital adjustment perceived by each spouse. According to Spanier (1976), the marital adjustment can be conceptualized as a process, which develops along a continuum; for this reason, it is possible to assess the dyadic adjustment at each stage of the investigation. The sum of the 32 items gives a total score indicating the individual perceived marital adjustment, which can vary from 0 to 151, with higher scores representing a better adjustment. The DAS showed high reliability at each stage of the present study: Cronbach’s alpha ranged between 0.84 and 0.92 for fathers, and 0.85 and 0.92 for mothers. As well as the other instrument, after administrating the questionnaire to both parents, an overall measure of the couple perception of marital adjustment was calculated if no statistical differences were found between mothers and fathers’ reports.
– The *LTP Procedure* (Fivaz-Depeursinge and Corboz-Warnery, 1999) is a semi-standardized role-play situation available in two versions, prenatal and postnatal; it has the aim to assess family interactive competences during different stages of child development. The prenatal LTP was administered at seventh month of pregnancy, since a representation of an imaginary baby is progressively elaborated during pregnancy by parents, peaking around the sixth month of pregnancy. The postnatal LTP was administrated at different times: 4th month (when the triangular interactive patterns, emerged during the previous months, showing a certain stability), 9th (which coincides with the emergence of child’s secondary intersubjective competences (Trevarthen and Hubley, 1978), 18th (corresponding to toddlerhood), and 36th/48th months of child’s life (the initial phase of the preschool age). The postnatal LTP is a play situation that lasts on average 15 min where father, mother, and their baby are together. Parents sit in front and at each side of their child, who sits between them; parents and child’s body positions thus form a triangle. The family is asked to interact playing together (with or without toys and objects, according to the child’s age) in a spontaneous manner, as they usually do at home. The play is structured in four parts, related to the four possible relational configurations that may occur in a triad: in the first one (2 + 1) one parent interacts actively with the child, while the other one observes; in the second one (2 + 1) the other parent plays the active role; the third part (3) shows parents and child playing all together; in the last part (2 + 1) parents talk to each other while the child takes up the third party position. The interaction is recorded with two cameras: one records the parents, while the other is set to record the baby. This observational tool is made up of ten assessment scales, rated on a 1–5 Likert scale (FAAS 4.0; Lavanchy-Scaiola et al., 2006, unpublished). The scales of FAAS 4.0 are: *Postures*: the basic level of interactions; it describes the “readiness to interact” signals and indicates the engagement in the interaction; *Gaze orientation*: mutual gaze orientation among family members; *Inclusion of partners*: the reciprocal interpersonal engagement within the group as a whole; *Support and cooperation between parents (co-parenting):* the support parents give/offer one another; *Implication of each partner in his/her role*: the position by which individuals modulate their involvement without breaking out of the interaction; *Parental scaffolding*: parents’ supervision of the child and appropriate stimulation to keep him/her engaged; *Infant’s involvement*: extent to which the child’s signals are clear and interpretable by the parent; *Co-construction:* inter-attentiveness, that is sharing a common object of attention through the orientation of the gaze or a common subject of discussion; *Sensitivity*: empathic emotional reactions, or sensitivity; *Family warmth*: the emotional characteristics that are most favorable to interaction, associated with optimal child development (McHale and Rasmussen, 1998). Every scale is assessed in each of the four procedure parts: the scores of each part (range: 10–50) are summed up, to obtain a global score (range 40–200) (Favez et al., 2011). The application of the LTP in the present research showed a good inter-rater reliability for each stages, ranging from *r* = 0.79 to *r* = 0.93. The overall internal consistency also was high (α = 0.96), ranging from 0.91 to 0.99 for the different stages.
– The *Prenatal LTP procedure* (Carneiro et al., 2006) is a semi-standardized role-play situation, developed on the methodological scheme of the postnatal LTP (it consists of four parts too), but it lasts about 5 min. Here, mother and father are involved with a “neutral” doll, which represents the baby, with the typical size and shape of a newborn; such “neutrality” should help parents-to-be to role-play the situation. The facilitator asks the parents to imagine the moment when the three of them will meet for the first time after delivery. The prenatal co-parenting interaction is assessed using five scales, each one ranging from 1 to 5 on a Likert Scale. The behavioral dimensions are: *Co-Parent Playfulness* that assesses a couple’s capacity to create a playful space and to co-construct a game; *Structure of the Play* that assesses the couple’s capacity to structure the four play segments according to instructions; *Intuitive Parenting Behaviors* encompasses holding and “en face” orientation, dialog distance, baby-talk and/or smiles at the baby, caresses and/or rocking, exploration of the baby’s body, and preoccupation with the baby’s well-being; *Couple’s Cooperation* that assesses – at behavioral level – the degree of active cooperation between the parents during the play; *Family Warmth* that captures the affection and humor shared by the partners during play; namely, whether they manifest affection and tenderness as a couple and toward the “baby.”

The total score of the prenatal LTP ranged from 5 to 25. In order to compare the prenatal and postnatal procedures, the prenatal total score was converted so that it could range from 40 to 200.

For the Prenatal LTP, the internal consistency was good (Cronbach’s α = 0.75). With respect to the inter-rater reliability, a good level emerged, namely *r* = 0.77.

Both LTP and Prenatal LTP video-recorded procedures were independently coded by trained and reliable judges, who were blind to the aims of the study; for each family, interactions of each developmental stage were rated by different coders and independently from their chronological sequence.

## Analytic Strategy

### Preliminary Analysis

For preliminary analysis, we compared mothers and fathers’ reports on marital satisfaction and paternal involvement in order to identify an overall index for each couple, in case no statistical differences emerged between parents. A correlated-samples *t*-test revealed no statistical differences in father involvement at any steps of the study. Moreover, significant Pearson’s correlations between mothers and fathers’ reports were reported to be good at each stage, ranging from 0.71 to 0.82. Similar results emerged for marital satisfaction, with no significant differences revealed by the *t*-test analysis and significant positive correlational values, ranging from 0.45 to 0.74.

Applying a repeated-measures ANOVA, we investigated the trend of father involvement from the fourth month of child’s life up to the preschool age. No statistical differences among different stages resulted in the levels of father involvement as perceived by parents (*F* = 0.41, *p* = 0.80). Moreover, Pearson’s correlations between the stages was presented as significant and ranged from 0.62 to 0.73.

### Multilevel Analysis: Growth Model

In the present model, the following variables were considered:

– *Dependent Variable*: Quality of triadic family interactions assessed by the LTP, at seventh month of pregnancy (prenatal LTP) and at 4th, 9th, 18th, and 36th/48th month of child’s life (postnatal LTP).
– *Within family predictors*: Marital quality assessed at each time point with the DAS;
– *Between family predictors*: Two socio-demographic variables were included in the analysis: child gender (0 = male, 1 = female) and the duration of the relationship between parents. Moreover, father involvement, investigated by the *Questionnaire on Father Involvement*, was included as a third “between-family” variable, since it resulted in a stable characteristic.
– Time was coded in linear form (0, 1, 2, 3, 4).

Given the multilevel nature of these data (which varied in terms of time and families), two-level hierarchical regression models were run using the Hierarchical Linear Modeling software (HLM, Raudenbush and Bryk, 2002). The within family influences exerted over time by time, and DAS were modeled at level 1:

$$Y_{ti}=\pi_{0i}+\pi_{1i}(time)+\pi_{2i}\left(DAS\right)+e_{ti}$$

In this equation, *t* is the index for observation occasions and *I* is the index for families. We considered our DAS variable as time-variable predictor, which may change over time. The intercept, π_0i_ represented the expected mean LTP for the *i*th family at time 0. π_1i_ and π_2i_ account for the change of LTP, respectively, due to time and DAS for the *i*th family. Finally, *e*_ti_ represented the random effect for the intercept and slopes. Based on Raudenbush and Bryk (2002), we entered these predictors into our equation as centered variables.

Lausanne Trilogue Play variations between families were modeled at level 2. The intercept at level 1 became the outcomes we tried to explain at level 2:

$$\pi_{0i}=\beta_{00}+\beta_{01}(gender\,\;child)+\beta_{02}(relationship)+\beta_{03}\left(father\,\;invo\,\mathrm{lm}ent\right)+r_{0i}$$

In this equation, β_0_’s represents the impact of the family level variables we used (child gender, relationships, father involvement) on the mean. The random effect for the intercept is represented by *r*_0i_.

The variability of the slopes was verified and no variability has been found.

The model was run in three steps. First, the unconditional model was run. The second step (Model 1) included the within-families (between time) variables: time and DAS. In the third step (Model 2) we added our predictors at the family level.

The multilevel analyses of longitudinal data enables to handle missing data (Snijders, 1996); more accurately, this refers to the ability to handle models with varying time points. Multilevel regression model does not assume equal number of observations (or even fixed time points), so respondents with missing observations (in the dependent variable) pose no special problems, and in all cases, at least two data points were present per family in the current analyses^2^.

## Results

**Table 1** reports the descriptive statistics for the variables we entered. Before running the multivariate models described above, an unconditional model was run. This model aimed to examine the variance of LTP, partitioning it into within-family/between-time and between-family variances. In our sample, 80.4% of the variation in LTP lied at the within-family level (between time), 19.6% was between families. Thus, according to the unconditional model in our dataset there was much greater variability within family between times than between families, indicating that the quality of family interaction assessed by LTP was not stable over time.

**Table 1:** Within family, between families variables: descriptive statistics.

Variables	Mean	*SD*	Min	Max
**Within Family level**				
*Time 0 (pregnancy)*				
Dyadic Adjustment Scale (*N* = 96)	121.96	9.39	98.00	143.50
Prenatal Lausanne Trilogue Play (*N* = 96)	140.33	33.30	64.00	192.00
*Time 1 (4th month)*				
Dyadic Adjustment Scale (*N* = 83)	120.84	110.74	88.08	142.50
Lausanne Trilogue Play (*N* = 87)	147.49	28.02	84.00	191.00
*Time 2 (9th month)*				
Dyadic Adjustment Scale (*N* = 75)	116.79	14.130	75.50	145.00
Lausanne Trilogue Play (*N* = 76)	162.18	21.54	75.00	195.00
*Time 3 (18th month)*				
Dyadic Adjustment Scale (*N* = 43)	114.83	15.69	79.50	143.50
Lausanne Trilogue Play (*N* = 40)	165.47	15.30	133.25	196.00
*Time 4 (preschool)*				
Dyadic Adjustment Scale (*N* = 47)	117.92	14.70	71.50	145.50
Lausanne Trilogue Play (*N* = 41)	162.68	24.59	86.00	196.00
**Between Families level (N = 103)**				
Child Gender (1 = female) %	59.80 (0)	40.20 (1)	0.00	1.00
Relationship (years)	7.91	4.40	2.00	24.00
Father Involvement	61.29	18.96	7.47	93.00

The core of the study lays in the examination of the influence of time on triadic interactions.

Analysis showed that time was positively related to the dependent variable; on the other hand, the effect of DAS was negative (Coefficient = -0.32, *p* = 0.05). Both variables explained 26.02% of the variability within family. Thus, our Model 1 showed that LTP, that is the mother–father–child interactions, significantly increases according to time and decreases according to DAS (**Table 2**).

**Table 2:** Correlates of Lausanne Trilogue Play.

	Unconditional Model				Model 1				Model 2			
Variables	Coefficient	*SE*	*t* ratio	*p*	Coefficient	*SE*	*t* ratio	*P*	Coefficient	*SE*	*t* ratio	*p*
Intercept	153.77	1.92	80.29	0.001	153.24	2.09	73.37.84	0.001	153.37	1.95	78.51	0.001
*Level 1 – within family (N = 310)*												
Time					3.50	0.74	4.71	0.001	3.53	0.74	4.78	0.001
Dyadic Adjustment Scale (DAS)					-0.31	0.18	-1.92	0.050	-0.32	0.17	1.95	0.050
*Level 2 – between families (N = 82)*												
Child Gender (1 = female)									0.03	3.82	0.01	0.993
Relationship									-0.56	0.36	-1.53	0.130
Father Involvement									0.34	0.17	3.09	0.003

**Variance components for π_0i_**	**Variance**	***SD***	**χ^2^**	***p***	**Variance**	***SD***	**χ^2^**	***P***	**Variance**	***SD***	**χ^2^**	***p***

Within family	143.30	11.97			236.52	15.38			207.30	14.40		
Between families	587.81	24.24	156.66	0.001	434.87	20.85	204.50	0.001	434.21	20.84	185.82	0.001

In Model 2, we added the between-families independent variables to predict LTP score – expected mean and modification connected to time – as a function of child gender, relationship, and father involvement. Consistent with our hypothesis, in families with higher level of father involvement the latter was able to predict high levels of our dependent variable.^3^ (**Table 2**).

## Discussion

The first purpose of the study was to investigate the trajectory of triadic family interactions, as assessed with LTP, father involvement and marital satisfaction from pregnancy to the preschool age. Consistent with our hypothesis, results showed that the quality of family interactions increased as a function of time and showed a developmental trend. These results do not support the stability model (Carneiro et al., 2006; Favez et al., 2006a), but they are in line with previous empirical evidence attested on an Italian sample (Bighin et al., 2011).

Different explanations can be taken into account in order to discuss the present data indicating an improvement of family interactions over time. First, the development of child interactive triadic abilities may contribute to the improvement of family interaction quality, since the infant becomes a more active and competent partner in the family interactions. Secondly, partners who daily experience repeated interactive exchanges develop a “relational” competence and a better knowledge of other partners’ usual behaviors, cues and intentions, fostering a virtuous cycle of the quality of family interactions. Eventually, from a methodological point of view, a possible reason for divergent results on the temporal trend of family interactions assessed using LTP may be detected in the use of two different methods of measurement. The first one, applied by the Lausanne Group (Carneiro et al., 2006; Favez et al., 2006a), is categorical and requires to categorize family interactions in four types of family alliance; the other one, used in the Italian sample (Bighin et al., 2011; Simonelli et al., 2014), is ordinal and assigns a score to interaction quality. Specifically, categorical data reduce the possibility to identify differences among measurements, whilst increasing the stability or lack of difference.

With regard to the trajectory of father involvement over time, the present study supported a stability model of the involvement of fathers in childcare; this can be explained by the fact that the global levels of father involvement in this study are generally high, or at least sufficient. In fact, comparing our results with data provided by Frascarolo (2004), it emerged that fathers in our group display similar high levels of involvement, showing that they take part in childcare in an adequate way and they steadily do it from the early postnatal life until the preschool age. Thus, a further improvement of this construct is neither expected nor necessary, as it would be in groups with initial low levels of father involvement. Concerning the marital adjustment measured with DAS, our data are consistent with a vast literature (Belsky et al., 1983; Terry et al., 1991; Favez et al., 2012; Kohn et al., 2012; Trillingsgaard et al., 2014) reporting a general decline in dyadic satisfaction over the transition to parenthood; specifically, this decline persists at least until 30 months after child’s birth. The present study contributes to extend the available results, attesting to the decrease of marital adjustment during the preschool age as well.

The second aim of our research was to analyze the longitudinal and multilevel effects exerted on the changes of interaction quality by two families of variables, respectively, lying at the within-family (between time) and at the between-families.

The within-family variable marital satisfaction, measured with DAS, resulted to be a determinant factor for the developmental trend of family interactions assessed by LTP; specifically, a decrease of marital adjustment perceived by partners over the transition to parenthood and until the preschool age is associated to an improvement of the quality of family interactions in this period. The current research supports the existing literature on the link between DAS and LTP; although scarce, it indicates a negative association between these two dimensions of family functioning (Favez et al., 2006b, 2011). This inverse relationship can be explained as a necessary and adaptive process; it is possible to suggest that over the transition to parenthood and in the following postnatal period, partners become less focused on their couple dynamics, in favor of dedicating more resources and attention to the father–mother–infant interactions, allowing the establishment of a new and adaptive triadic family system. A methodological consideration on the negative association reported between DAS and LTP might concern the different assessment measures applied, the one self-report (DAS), the other observational (LTP).

With regard to the between-families variable, Father Involvement in childcare, results showed that its levels predicted the quality of family interactions from the earliest stages of child’s life. In particular, higher levels of Father Involvement reported by parents corresponded to better interactive competences of the family during the triadic play situation. Moreover, these results indicated that the levels of father involvement can be considered as a family characteristic which arises in the earlier stages of the postnatal period and shows a stable trend, rather than developing during the course of the first years of child’s life, in response to different baby’s developmental needs and abilities, or subsequently to an enhanced reciprocal relational knowledge among family members. As a consequence, it is possible to argue that father involvement in childcare can be conceived as an early resource for the family system; it may constitute a background which provides family members with stable and specific relational competences, upon whom partners can rely on, in interacting with each other over infancy. These results are in line with studies that underline the positive effect of high levels of father involvement on child development, mother– and father–child relationships and the marital subsystem (Venuti and Giusti, 1996; Frascarolo, 2004; Pleck and Masciadrelli, 2004; Sarkadi et al., 2008).

From an operational point of view, the main implications of the present results point out that a decrease in marital satisfaction may represent an expected and not problematic event in family life cycle; moreover, it is counterbalanced by an increase in mother–father–child interactions. At the same time, preventive programs which aim to help parents to reinforce their conjugal relationship might be useful in order to support the marital couple throughout the transition to parenthood and to buffer the effects of a too steep decline. Secondly, given the crucial role played by father involvement as a stable factor predicting the quality of family interactions, protocols fostering fathers’ role should be ideated and implemented at a community level.

### Strengths, Limitations, and Future Directions

Our research has some strong points, mainly considering its longitudinal and growth model approach. Moreover, concerning the quality of the information collected, the use of an observational procedure to investigate family exchanges is noteworthy; it allows a direct and standardized measurement of family processes, overcoming the limits inherent to the self-report assessment. Thus, the present study takes the advantage of multi-method approach, integrating self-report and observational measure. The number of participants and their Italian origin represent other strengths of the current research; our sample size exceeds the number of family commonly involved in most studies on early family interactions. Lastly, from a broader perspective, this study can offer a valuable contribution to the study of family systems, since it considers simultaneously, three different constructs (i.e., father involvement, dyadic satisfaction, and family interactions), frequently investigated independently.

However, it also had some limitations. First, our sample is composed of families that attended child’s birth preparation classes and voluntarily participated in the study, thus it cannot be considered representative from a statistical point of view and further research should be based on stratified samples. Second, a s*elf*-*selection* bias might have arisen, since families who were motivated to participate in all the stages of the study might be those who invest more on the family system and, thus, display a better quality of family interactions and dynamics. Moreover, even with the use of a random effects approach, the level of attrition remains high and, consequently, restricts the generalizability of the present results.

Another limitation of the research can be detected in the absence of specific measures of child’s temperament, development and competences, mainly from the interactive point of view, suggesting new directions for further studies. Finally, with reference to Belsky’s (1984) model of parenting, the present study does not include other important factors, besides child normative and clinical characteristics detected by early assessment (Simonelli, 2013). Other factors include contextual sources regarding parent’s work and social support, and other adults’ variables, such as personality. Similarly, future directions could look at reciprocal influences between marital quality and father involvement, as so as the effect of family interactions on couple adjustment.

In addition to the investigation of the potential influence of the above-mentioned factors on the family processes, a further future direction of this study might be the adoption of a wider longitudinal perspective, until child’s school age.

## Author Contributions

AS, MP, CS, FD, and AV have given a substantial contribution to the conception and implementation of the work, taking part to data acquisition, analysis and discussion, drafting and revising the manuscript. All authors revised and reached an agreement on the final version of the work.

## Conflict of Interest Statement

The authors declare that the research was conducted in the absence of any commercial or financial relationships that could be construed as a potential conflict of interest.

## Footnotes

1 This study was carried out in accordance with the recommendations of the Code of Ethics approved by the General Assembly of the Italian Association of Psychology. Parents signed an informed consent form declaring their consent and authorizing to use personal data and recordings for the purpose of research and indicating their approval for their child to have their child participate, according to the law on privacy.

2 Due to incomplete response on the final two assessments, parallels analyses has been conducted by removing the final two time points, obtaining similar results.

3 We controlled for the variability of the slope. The effect of time was significantly different between families [χ^2^(69) = 100.15, *p* = 0.009] but none of the families predictors explained this variability. The effect of DAS resulted invariant [χ^2^(69) = 74.33, *p* = 0.309].
